# Regulation of Hepatocyte Nuclear Factor 4α Attenuated Lipotoxicity but Increased Bile Acid Toxicity in Non-Alcoholic Fatty Liver Disease

**DOI:** 10.3390/life12111682

**Published:** 2022-10-22

**Authors:** Yoon Jin Roh, Yun Kim, Jae Sun Lee, Ju Hee Oh, Seung Min Lee, Eileen Laurel Yoon, Sung Ryol Lee, Dae Won Jun

**Affiliations:** 1 Department of Dermatology, Chung-Ang University Hospital, Seoul 04763, Korea; 2 Hanyang Medicine-Engineering-Bio Collaborative & Comprehensive Center for Drug Development, Hanyang University, Seoul 04763, Korea; 3 College of Pharmacy, Daegu Catholic University, Gyeongsan 38430, Korea; 4 Department of Translational Medical Science, Hanyang University Graduate School of Biomedical Science and Engineering, Seoul 04763, Korea; 5 Department of Gastroenterology, Hanyang University School of Medicine, Seoul 04763, Korea; 6 Hanyang Institute of Bioscience and Biotechnology, Hanyang University, Seoul 04763, Korea; 7 Department of Surgery, Kangbuk Samsung Hospital, Sungkyunkwan University School of Medicine, Seoul 03181, Korea

**Keywords:** hepatocyte nuclear factor 4 alpha, non-alcoholic fatty liver disease, lipotoxicity, bile acid toxicity, steatohepatitis

## Abstract

**Simple Summary:**

Hepatic HNF4α inhibition can attenuate bile acid toxicity in NAFLD. However, it can aggravate hepatic steatosis, so it is necessary to study the optimal timing of HNF4α inhibition and the development of surrogate markers that can predict it. It is known that bile acid–induced toxicity, along with lipotoxicity, plays a very important role in NAFLD patients. Except in very exceptional conditions, lipotoxicity caused by free fatty acids and bile acid toxicity caused by bile acid always coexist in the NAFLD human condition. In this respect, we believe that inhibiting HNF4α is more effective as a therapeutic strategy for NAFLD.

**Abstract:**

Hepatocyte nuclear factor 4 alpha (HNF4α) is a key master transcriptional factor for hepatic fat and bile acid metabolic pathways. We aimed to investigate the role of HNF4α in non-alcoholic fatty liver disease (NAFLD). The role of HNF4α was evaluated in free fatty acid–induced lipotoxicity and chenodeoxycholic acid (CDCA)-induced bile acid toxicity. Furthermore, the role of HNF4α was evaluated in a methionine choline deficiency (MCD)-diet-induced NAFLD model. The overexpression of HNF4α reduced intracellular lipid contents and attenuated palmitic acid (PA)-induced lipotoxicity. However, the protective effects of HNF4α were reversed when CDCA was used in a co-treatment with PA. HNF4α knockdown recovered cell death from bile acid toxicity. The inhibition of HNF4α decreased intrahepatic inflammation and the NAFLD activity score in the MCD model. Hepatic HNF4α inhibition can attenuate bile acid toxicity and be more effective as a therapeutic strategy in NAFLD patients; however, it is necessary to study the optimal timing of HNF4α inhibition.

## 1. Introduction

Non-alcoholic fatty liver disease (NAFLD) has become the most common cause of chronic liver disease. NAFLD includes wide spectrum diseases ranging from simple steatosis, non-alcoholic steatohepatitis (NASH), fibrosis, to liver cirrhosis [1].

Hepatocyte nuclear factor-4α (HNF4α) is a nuclear receptor superfamily which plays an important role in early hepatocytes development [2]. HNF4α is a master regulator of hepatic genes’ expression for fat and bile acid metabolic pathways [3,4]. HNF4α is known to play an important role in the production of bile acids and the excretion of fatty acids in the hepatocyte [5,6]. HNF4α is known to increase bile acid production via an increase in the activity of Cyp7a1 [6]. However, at the same time, HNF4α reduces fat accumulation via promoting the excretion of fatty acids from hepatocytes. The promotion of intracellular fatty acid excretion by HNF4α might have a positive role in reducing cell lipotoxicity, but it might also have a negative role in increasing bile acid toxicity by increasing bile production. NAFLD is a disease with pathophysiology, where both lipotoxicity and bile acid toxicity are important. For this reason, the role of HNF4α in NAFLD is still largely complex and unknown.

So far, data on the association between HBF4 and HNF4α are very diverse. Generally, HNF4α has been shown to have protective effects on hepatis steatosis. Liver-specific HNF4α ^−/−^ mice showed severe fatty liver in association with disruption of very low density lipoprotein (VLDL) secretion [2,7]. In humans, HNF4α loss-of-function mutations were associated with maturity-onset diabetes [8]. However, HNF4α seems to have a positive correlation with hepatic and systemic inflammation. Hepatic HNF4α expression increased in NAFLD compared to the control [9]. Yu et al. showed cytoplasmic retention of hepatic HNF4α cause increasing hepatocellular inflammation in high-fat-diet-fed mice [10]. Moreover, another study also suggested that HNF4α increases endoplasmic reticulum stress response [11].

Taken together, the effects of HNF4α on hepatic fat accumulation and hepatic inflammation look different. Thus, our current understanding regarding the role of HNF4α is still complex in NAFLD. We tried to investigate the comprehensive effects of HNF4 on intrahepatic fat, inflammation, bile acid metabolism, and cell death in NAFLD.

## 2. Materials and Methods

### 2.1. Hepatic HNF4α Expression in NAFLD Patients

Hepatic HNF4α expression was compared between thirty-three NAFLD patients and sixteen healthy controls. NAFLD was defined as the case of more than 5% of hepatocytes in which fat deposition was observed in liver tissue. Moreover, viral hepatitis A, B, and C; drug-induced hepatitis; alcoholic and/or autoimmune liver disease were excluded. Patients with alcohol consumption of >210 g/week for men and >140 g/week for women were also excluded from NAFLD. For the control group, normal liver tissue was obtained from 16 patients through hepatic resection because of benign disease. All control subjects had normal liver enzymes and <5% hepatic fat content. The study was approved by the institutional review board at Hanyang University Hospital. The protocol was registered at the Clinical Research Information Service (http://cris.nih.go.kr/cris/index.jsp; accessed on 26 September 2022) with the registration number KCT0000900. The patients consented to use the samples for research purposes.

### 2.2. Hepatic HNF4α Expression NAFLD Animal Models

Hepatic HNF4α expression was evaluated in two types of animal NAFLD models (24 weeks high fat (HF)-diet and 14 weeks methionine and choline deficiency (MCD)-diet models). All procedures were approved by the Hanyang Institutional Animal Care and Use Committee (HY-IACUC-11-067).

### 2.3. Histology Assessment

To assess hepatic steatosis, liver tissue sections were scored for activity (degree of inflammation) and stage (degree of fibrosis) of disease according to the histological grading and staging systems, respectively. Hepatic steatosis was graded as follows: <5% (score, 0), 5–33% (score, 1), >33–66% (score, 2), and >66% (score, 3); steatosis, 0–3; lobular inflammation, 0–4; porto-periportal activity, 0–4; and fibrosis, 0–4. For NAFLD, the histological changes were graded by using the NASH clinical research network scoring system [12]. A single blinded pathologist evaluated histological characteristics and calculated overall steatohepatitis scores.

### 2.4. Immunohistochemistry

Formaldehyde-fixed (10%) and paraffin-embedded liver tissue sections were stained with hematoxylin and eosin. Four-micrometer-thick sections were cut from paraffin block and coated on a glass slide. Immunohistochemistry for HNF4α, NTCP, and Cyp7a1 (Abcam, Cambridge, UK) expressions (Abcam, Cambridge, UK) was performed. A single-blinded pathologist evaluated histological characteristics, intensity, extent, and immune-reactive scores of immune-stained sections.

### 2.5. shHNF4α Construction and Animal Study

Ad-shHNF4α and Ad-empty (control) were constructed. When Ad-HNF4α was constructed, only the HNF4α coding region (which does not include any 3′UTR) was cloned into the adenoviral vector. Ad-shHNF4α and Ad-empty (control) were generated by cloning the HNF4α coding region into a pAdEasy-1 vector (catalog #240005, Agilent), followed by transfection into HEK 293 cells for adenovirus production. Cells were infected with adenoviruses at an MOI of 10. Mice were intravenously (via tail vein) injected with 2.5 × 10^6^ pfu adenoviruses. The viral titers (plaque-forming units) were determined in HEK293 cells, using standard procedures. Comparable amounts of Ad-RGD were used as a control. The use of animals in this work was in accordance with the Hanyang Institutional Animal Care and Use Committee. Forty-five male C57BL/6 mice were randomly divided into the following groups: control (*n* = 15) fed on MCD diet, and Sh-HNF4α (*n* = 15) fed on MCD diet with Ad-shHNF4α injection for 14 weeks. Animals were maintained in a temperature-controlled room (22 °C), on a 12:12 h light–dark cycle. Tail-vein injection was performed by using a 1 mL insulin syringe with 28 gauge. Mice were given daily tail-vein injections with saline, control shRNA, or shHNF4α for 4 weeks, from the 10th week to 14th week. Mice were restrained for a single 300 μL tail-vein injection of either saline, 2.5 × 10^12^ viral particles of adenovirus expressing nontargeting control shCon, or 2.5 × 10^12^ viral particles expressing shRNA targeting HNF4α. The body weights of mice were assessed weekly. Four weeks post-injection, animals were humanely euthanized by CO_2_ inhalation, and plasma and liver samples were collected for analysis.

### 2.6. HNF4α Overexpression in In Vitro System

The coding regions of the human HNF4α gene from TrueORF cDNA Clones (Origene, Rockville, MD) were amplified by using PCR. The fragment was cloned into pECFP (enhanced green fluorescent protein)-C1 vector (Clontech, Palo Alto, CA). To generate the HNF4α construct, PCR products were subcloned into pGEM-T easy vector (Promega, Madison, WI) and then cloned into EcoRI-BamHI sites of the pECFP-C1 vector. For HNF4α overexpression, electroporation was performed with Microporator (Thermo Fisher Scientific, Waltham, MA USA), using the manufacturer’s recommended settings (1400 voltage, 20 ms, and two pulses). For quantitative measurement of transfection efficiency, the percentage of transfected cells was measured by using the LightCycler 480 system^®^ (Roche Diagnostics, Mannheim, Germany) after electroporation.

### 2.7. siRNA Knockdown

For HNF4α knockdown, HepG2 cells were transfected with Silencer predesigned human HNF4α siRNA and Negative Control #1 siRNA (Thermo Scientific, Waltham, MA, USA), using Lipofectamine RNAi/MAX transfection reagent (Invitrogen, Carlsbad, CA, USA), following the manufacturer’s instructions. Two days after siRNA transfection, the cells were collected for analysis.

### 2.8. Cell Culture

HepG2 cells obtained from American Type Culture Collection (Rockville, MD) were grown in Dulbecco’s modified Eagle medium (DMEM, Invitrogen, Carlsbad, CA, USA) supplemented with 10% fetal bovine serum and penicillin (50 U/mL)/streptomycin (50 µg/mL) (Invitrogen).

### 2.9. Western Blot Analysis

Liver tissue samples were solubilized in radioimmunoprecipitation assay buffer containing protease inhibitors (Pierce, Rockford, IL, USA). The lysates were centrifuged (13,000× *g* for 10 min at 4 °C), and supernatants were boiled with sodium dodecyl sulfate buffer (0.5 M β-mercaptoethanol). Later, the lysates were subjected to 12% SDS–PAGE for Western blot analysis. Reactive protein bands were analyzed by enhanced chemiluminescence detection reagent (Amersham Biosciences, Piscataway, NJ, USA), using an image reader LAS-3000 (version 2.1; Fujifilm, Tokyo, Japan). HNF4α polyclonal antibodies (Santa Cruz Biotechnology, Inc., Santa Cruz, CA, USA) were diluted 1:500 in TBS-T/5% BSA. Horseradish peroxidase (HRP)-conjugated goat anti-rabbit IgG antibody diluted 1:2000 (Santa Cruz Biotechnology, Inc.) in TBS-T/5% BSA was used as a secondary antibody. The experiment was repeated using three different samples.

### 2.10. TUNEL Assay

The TUNEL reaction was carried out by using the “In situ cell death detection kit, fluorescein” (Roche, USA) according to the manufacture’s instruction. Confocal imaging was performed by using a Leica TCS SP5 confocal microscope (Leica Microsystems, Wetzlar, Germany).

### 2.11. MTT Assay

MTT [3-(4,5-dimethylthiazol-2-yl)-2,5-diphenyl tetrazolium bromide] was purchased from Sigma-Aldrich (Saint Louis, MO, USA). HepG2 cells were seeded onto 96-well plates (5000 cells/well), using DMEM. After 24 h, the cells were incubated with palmitic acid (PA; 400 μM), chenodeoxycholic acid (CDCA; 100 μM), a combination of CDCA and PA, and DMSO (mock control) for 24 h. CDCA is one of the most toxic bile acids with hydrophobic profile that can induce hepatotoxicity [13,14,15]. After treatment incubation, MTT solution (0.25 mg/mL MTT in PBS, pH 7.4) was added to each well, followed by incubation at 37 °C for 4 h. The supernatants were removed from wells, and DMSO was added to dissolve crystals. The optical density was measured at 540 nm, using an ELISA microplate reader (Tecan, Research Triangle Park, NC, USA).

### 2.12. Statistical Analysis

All quantitative data were expressed as group means and standard deviations. Statistical analysis was performed with a Kruskal–Wallis test and Mann–Whitney’s U test, using SPSS for Windows version 18.0 (SPSS Inc., Chicago, IL, USA). The *p*-values less than <0.05 were considered significant.

## 3. Results

### 3.1. Hepatic HNF4α Expression Increased in NAFLD

**Figure 1:**
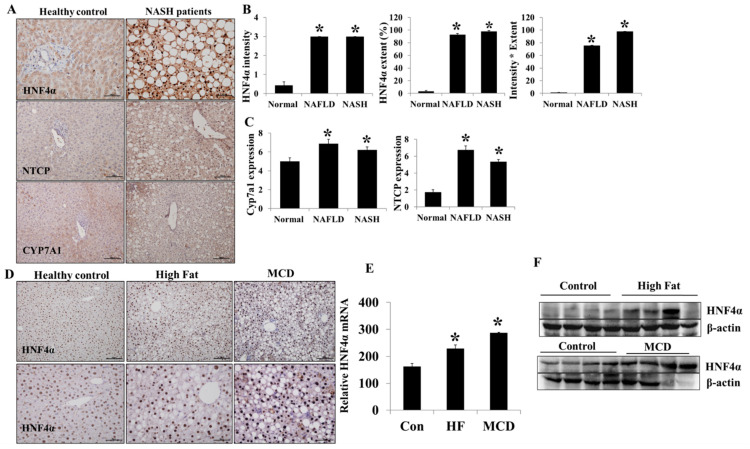
Hepatic HNF4α expression in NAFLD. (**A**) Hepatic HNF4α, NTCP, and Cyp7a1 immuno-expression in healthy controls and NAFLD patients. (**B**) HNF4α expression intensity, extent, and immune-reactive score (intensity × extent) in healthy controls, simple steatosis (NAFLD), and steatohepatitis (NASH) patients. (**C**) Hepatic NTCP and Cyp7a1 immuno-expression in healthy controls and NAFLD and NASH patients. (**D**) Hepatic HNF4α immune expression in HF-diet- and MCD-diet-induced animal NAFLD models. (**E**,**F**) Hepatic HNF4α mRNA and protein expression in HF-diet- and MCD-diet-induced animal NAFLD models. * *p* < 0.05 by Kruskal–Wallis test.

Hepatic HNF4α expression was evaluated in sixteen control and thirty-three NAFLD (seven simple steatosis and twenty-six steatohepatitis) patients (Figure 1A,B). Hepatic HNF4α expression was higher in NAFLD than in the control group (*p* < 0.05). However, HNF4α hepatic expression was not different between NAFLD and NASH groups. Hepatic HNF4α expression was higher in both NAFLD models (HF-diet and MCD-diet models) compared to the control (Figure 1D–F). The sodium/taurocholate cotransporting polypeptide (NTCP) and Cyp7a1 (cytochrome P450 family 7 subfamily A member 1) expressions were also higher in NAFLD compared to the control group (*p* < 0.05) (Figure 1A,C). The baseline clinical characteristics of the healthy controls and NAFLD patients are described in Table 1.

### 3.2. HNF4α Overexpression Attenuated Lipotoxicity

**Figure 2:**
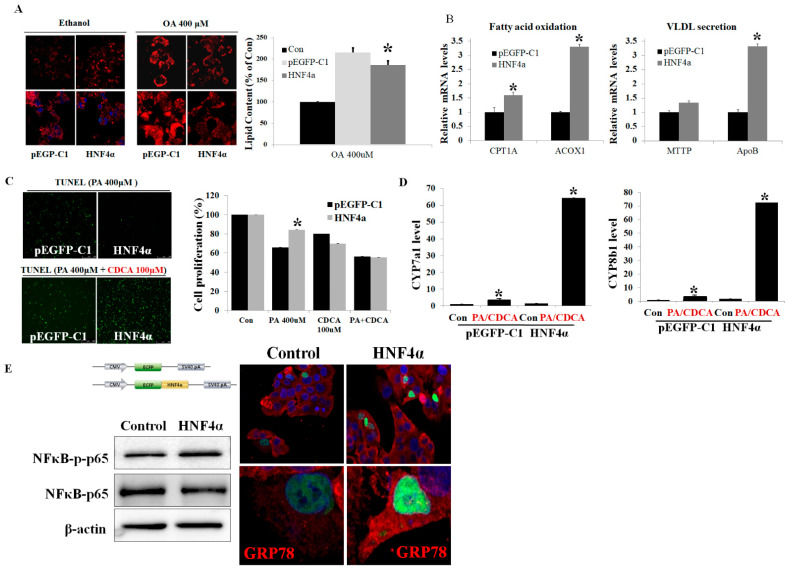
HNF4α overexpression attenuated lipotoxicity, but it deteriorated lipotoxicity in the presence of bile acid. (**A**) Nile-red staining in HepG2 cells transfected with HNF4α under ethanol and oleic acid (OA) pretreatment. (**B**) Effects of HNF4α overexpression on hepatic fat metabolism. (**C**) Effects of HNF4α overexpression on apoptosis (TUNEL staining) and cell proliferation (MTT assay) in PA-alone-treated and PA+CDCA-co-treated HepG2 cells. (**D**) mRNA expression of bile acid synthesis genes (Cyp7a1, and Cyp8b1) in PA+CDCA-co-treated HepG2 cells. (**E**) NF-κB-p-p65 protein expression and GRP78 immunofluorescence expression in HNF4α overexpressed HepG2 cells; p65 immunofluorescence expression in GFP-tagged HNF4α overexpressed HepG2 cells. * *p* < 0.05 by Kruskal–Wallis test.

Overexpression of HNF4α reduced intracellular lipid contents via increasing mitochondria beta-oxidation (CPT1A and ACOX1) and hepatic fat excretion (ApoB) (Figure 2A,B). PA treatment increased cell apoptosis (TUNEL satin) and decreased cell viability (MTT assay) (Figure 2C). PA-induced apoptosis decreased after HNF4α overexpressed HepG2 cells. HNF4α overexpression attenuated PA-induced cell death.

### 3.3. HNF4α Overexpression Increased NFκB via Activating Bile Acid Synthesis

The protective effects of HNF4α on lipotoxicity were reversed when CDCA was used in a co-treatment with PA (Figure 2C). HNF4α increased cell apoptosis rather than recovering cell proliferation under bile acid–induced toxicity (PA-and-CDCA co-treatment). CDCA treatment increased the rate-limiting enzymes (Cyp7a1 and Cyp8b1) for bile acid synthesis. HNF4α increased Cyp7a1 and Cyp8b1 mRNA expressions sharply, up to 60-folds (Figure 2D). HNF4α overexpression under CDCA activated the NFκB pathway. Double staining for GRP78 and p65 revealed that both signals were co-localized in the nucleus (Figure 2E). HNF4α increased intra-nuclear GRP78 and p65 expression.

### 3.4. Inhibition of HNF4α Attenuated Bile Acid Toxicity

**Figure 3:**
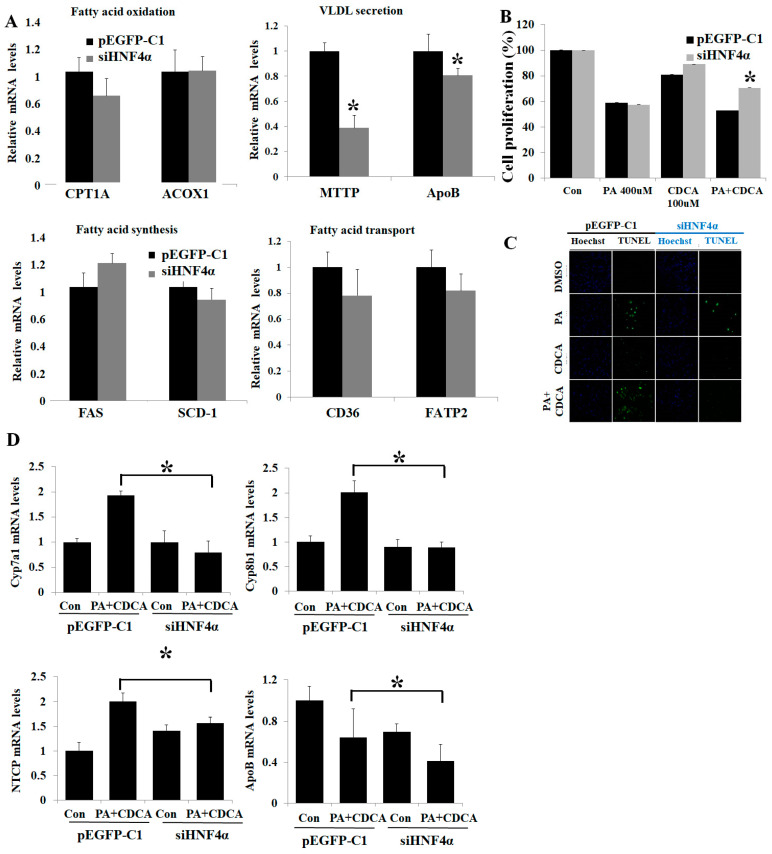
Inhibition of HNF4α attenuated lipotoxicity in NAFLD in vitro model. (**A**). Inhibition of HNF4α did not affect gene expression of hepatic lipid metabolism in HepG2 cell. (**B**) MTT assay of HepG2 cells after PA-and-CDCA co-treatment with/without HNF4α inhibition. (**C**). TUNEL assay on the HepG2 cells after PA-and-CDCA co-treatment. TUNEL-positive cells are stained in green, and all nuclei are stained in blue with DAPI. (**D**) Hepatic mRNA expressions of bile acid–related genes were measured by using RT-PCR. * *p* < 0.05 by Kruskal–Wallis test.

The inhibition of HNF4α by using small interfering RNA did not affect hepatic fat de novo synthesis and oxidation-related gene expressions (FAS, SCD-1, CD36, FATP2, CPT1A, and ACOX1). However, HNF4α inhibition decreased hepatic fat excretion–related gene expression (MTTP and ApoB) (Figure 3A). CDCA mono-treatment did not affect cell viability, but co-treatment with PA and CDCA decreased cell viability (Figure 3B,C). HNF4α knockdown using small interfering RNA recovered cell apoptosis and increased cell proliferation from the PA-and-CDCA-co-treatment condition (Figure 3B,C). HNF4α knockdown reduced bile acid uptake (NTCP) and bile acid synthesis (Cyp7a1 and Cyp8b1) (Figure 3D).

### 3.5. Inhibition of HNF4α Attenuated Hepatic Inflammation and Fibrosis in NAFLD Animal Model

**Figure 4:**
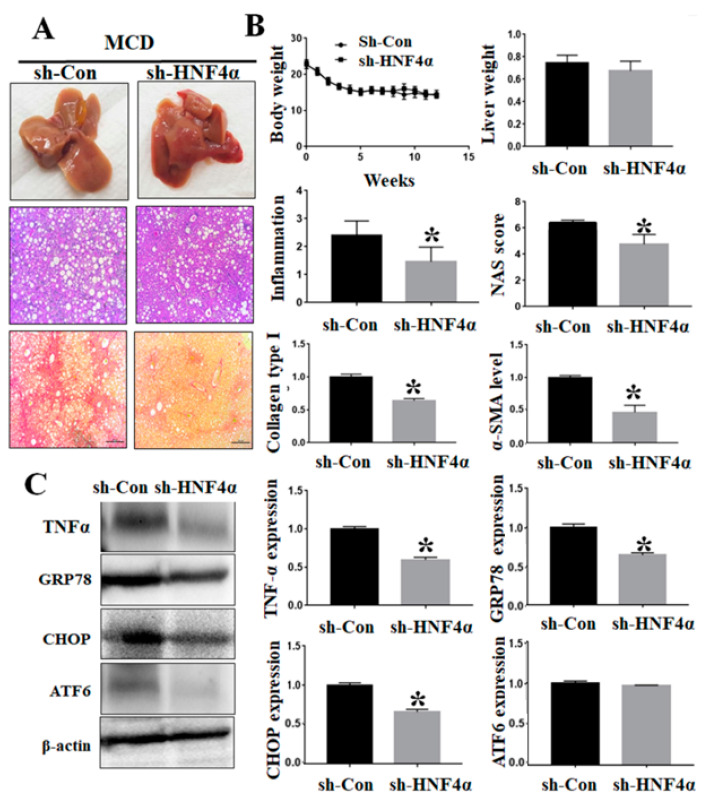
Effects of HNF4α inhibition in MCD-diet-induced NAFLD model. (**A**) Histological changes following sh-HNF4α adenovirus injection in NAFLD model. Liver tissue sections were stained with H&E and Sirius Red stains. (**B**) Changes in body weight and liver weight following sh-HNF4α adenovirus injection in NAFLD model. The extent of changes was quantified by using NAFLD activity score (NAS). RT-PCR analysis of liver tissue sections for TNF-α, GRP78, CHOP, and ATF6 expressions in sh-HNF4α and control groups. (**C**) Liver tissue TNF-α, GRP78, CHOP, and ATF6 proteins expression, using Western blot. * *p* < 0.05 by Mann–Whitney’s U test.

The sh-HNF4α and control adenovirus were injected into an MCD-diet-induced NAFLD model. Inhibition of HNF4α by using sh-HNF4α adenovirus vector did not have any effect on the body weights compared to the matched control group. The inhibition of HNF4α did not reduce hepatic fat accumulation effectively, but it decreased the intrahepatic inflammation and NAFLD activity score compared to the control (Figure 4A). Hepatic pro-fibrogenic markers such as α-SMA and COL1A1 were decreased in the sh-HNF4α group (Figure 4B). The sh-HNF4α group showed reduced intrahepatic mRNA expressions of TNF-α, GRP78, and CHOP compared to the control group. Moreover, hepatic endoplasmic reticulum stress markers (TNF-α, GRP78, CHOP, and ATF6 protein expressions) were significantly decreased in the sh-HNF4α group compared to the control group (Figure 4C).

## 4. Discussion

Our data showed that HNF4α expression was higher in NAFLD patients than healthy controls. HNF4α inhibition increased hepatic fat accumulation, but it attenuated steatohepatitis, as well as hepatic fibrosis, via reduction of bile acid toxicity.

Our results suggested that HNF4α overexpression decreased hepatic fat accumulation and attenuated free fatty acid–induced lipotoxicity. HNF4α overexpression upregulated fatty acid oxidation–related and fatty acid excretion–related genes’ expression. These findings were comparable with previous studies. The conditional liver-specific HNF4α disruption in mice led to severe steatosis and reduced serum triglycerides, which were associated with the selective disruption of ApoB and MTTP genes’ expression [2]. Other similar studies have shown that hepatic HNF4α deletion causes fatty liver in mice [2,7]. 

However, the role of HNF4α was complex in NAFLD. HNF4α is the master regulator of not only hepatic fat homeostasis, but also bile acid homeostasis. HNF4α and farnesoid X receptor (FXR) together coordinate to regulate bile acid synthesis. In Figure 3B, PA treatment significantly reduced cell proliferation, but CDCA did not. Interestingly, HNF4α siRNA treatment did not recover cell proliferation in the PA-alone treatment, but it did in the PA-and-CDCA-co-treatment group. It meant that it is already very well-known that PA treatment induces lipotoxicity and decreases cell proliferation. In terms of PA-induced lipotoxicity, overexpression of HNF4α has a protective effect on lipotoxicity. For this reason, there was no cytoprotective effect by HNF4α siRNA treatment in the PA alone. However, HNF4α plays an important role in the oxidation of fatty acids, but also in the synthesis bile acids. Overexpression of HNF4α has an effect of protecting lipotoxicity in the lipotoxicity condition caused by PA treatment, but inhibition of HNF4α is more effective in the NAFLD condition, where bile acid toxicity and lipotoxicity co-exist. In terms of bile acid homeostasis, HNF4α increases cholesterol 7α-hydroxylase (Cyp7a1) expression and increased bile acid toxicity [16]. The protective effects of free fatty acid–induced lipotoxicity were reversed under bile acid (or bile acid toxicity). The co-treatment of PA and CDCA without HNF4α overexpression did not increase Cyp7a1 and Cyp8b1 expressions. Cyp7a1, Cyp8b1, and NTCP mRNA expressions were 60-folds greater in HNF4α-overexpression groups co-treated with CDCA and PA. Lipotoxicity caused by free fatty acid must be an important key pathophysiologic pathway in NAFLD. However, in actual in vivo experiments, lipotoxicity caused by free fatty acids does not exist alone, and it always acts together with bile acid toxicity caused by bile acids. Therefore, we must consider both lipotoxicity and bile acid toxicity in understanding the pathophysiology of NAFLD.

Our results suggest that hepatic HNF4α expression is increased in NAFLD patients and animal NAFLD models. The hepatic HNF4α immunostaining was significantly increased in NAFLD and NASH patients. Moreover, hepatic HNF4α expression was also significantly increased in both HF-diet- and MCD-diet-induced NAFLD models. However, a previous study showed significantly reduced HNF4α expression in NASH patients [17]. It is unclear why the current results contrast with the previous study. There are several possible explanations. Firstly, when consider the different stages of disease progression in enrolled patients, HNF4α expression might be different according to the disease progression in NAFLD. Secondly, HNF4α expression might be related to the proportion of P1/P2 in the HNF4α ratio. HNF4α has two promoters (P1 and P2) and encodes at least twelve isoforms through differential splicing. Moreover, the P1/P2 promotor-related HNF4α ratio is partially controlled by the de-differentiated state [18].

Recent studies have shown increased toxic bile acids in liver tissues of NASH patients [19,20,21]. Our study is a study that reminds us, once again, of the importance of bile acid metabolism and bile acid toxicity in NAFLD. Several studies demonstrated that HNF4α is a critical transcriptional regulator of the Cyp7a1 gene [6,22,23]. Although we did not measure the actual bile acid levels in either humans or mice with and without NAFLD/NASH, the difference in bile acid profiles between NAFLD/NASH patients and healthy controls are reported [24]: serum total bile acid levels in NASH patients were approximately three times greater than those of healthy controls, and particularly deoxycholic acid was increased by four times (*p* < 0.001) in NASH patients. Moreover, the expressions of both CYP7A1 and CYP8B1, the rate-limiting enzymes of bile acid synthesis in the livers of these patients, were significantly increased in NASH patients [24]. As well as in the livers of HF-diet-fed rats, the expressions of Cyp7a1, Cyp27a1, FXR, and HNF4α were significantly increased compared to those in the controls [24]. Meanwhile, a putative HNF4A binding site (DR1) was identified in the murine Cyp7a1 gene promoter [25]. Taken together, increased expression of HNF4α in the liver of NASH patients supports the regulation of bile acids via upregulation of Cyp7a1 and Cyp8b1.

An interesting finding is that the CDCA mono-treatment did not affect cell viability, but the co-treatment with PA and CDCA decreased cell viability in our study. This finding was also reconfirmed from the other study [26]. Steatotic hepatocytes were shown to be more susceptible to bile acid–induced apoptosis [26]. Even low elevations of bile acids normally considered not harmful could enhance hepatocyte apoptosis in patients with NAFLD [27]. 

We are suggesting that hepatic HNF4α inhibition can attenuate bile acid toxicity and be more effective as a therapeutic strategy in NAFLD patients; however, it is necessary to study the optimal timing of HNF4α inhibition due to the worsening of hepatic steatosis. Moreover, due to their severe structural disorder and lack of clearly defined small-molecule binding sites, transcription factors have long been regarded as “undruggable” by small molecules [28,29]. Nevertheless, new therapeutics that target transcription factors are now becoming possible thanks to improvements in the understanding of the structure, function (expression, degradation, and interaction with co-factors and other proteins), and dynamics of the transcription factors’ manner of DNA binding [28,29].

Our study had several limitations. First, we should have shown the same contrast and intensity of DAPI in Figure 2A,C. We did not use the same positive control for imaging between groups. The DAPI intensity should be the same in all of the images. Second, we used an MCD model instead of the most widely used HF- or Western-diet model in this study, because we focused on the anti-inflammation and anti-fibrosis effect of HNF4α. Although there is still no satisfactory animal model in NAFLD, the physiology of the MCD model is somewhat different from obese NAFLD subjects.

## 5. Conclusions

In conclusion, hepatic HNF4α inhibition can attenuate bile acid toxicity in NAFLD. However, it can aggravate hepatic steatosis, so it is necessary to study the optimal timing of HNF4α inhibition and the development of surrogate markers that can predict it. It is known that bile acid–induced toxicity, along with lipotoxicity plays a very important role in NAFLD patients. Except in very exceptional conditions, lipotoxicity caused by free fatty acids and bile acid toxicity caused by bile acid always coexist in NAFLD human condition. In this respect, we believe that inhibiting HNF4α is more effective as a therapeutic strategy for NAFLD.

## Figures and Tables

**Table 1:** Baseline clinical characteristics of healthy controls and NAFLD patients.

Characteristics	Control (*n* = 16)	NAFLD (*n* = 33)	* *p*-Value
Sex (Men, %)	18.8%	48.5%	0.011
Age (year)	45.2 ± 13.2	45.3 ± 14.7	0.983
Height (cm)	160.7 ± 6.1	165.7 ± 10.5	0.044
Weight (kg)	59.6 ± 10.7	72.7 ± 12.6	<0.001
AST (IU)	29.9 ± 1.2	36.3 ± 18.9	<0.001
ALT (IU)	17.1 ± 7.1	51.9 ± 41.0	<0.001
Bilirubin (mg/dL)	0.6 ± 0.2	0.8 ± 0.7	0.423
Albumin (mg/dL)	4.5 ± 0.2	4.3 ± 0.4	0.067
Glucose (mg/dL)	97.8 ± 17.7	109.6 ± 52.7	0.295
GGT (mg/dL)	30.4 ± 23.2	57.5 ± 44.4	0.011
Cholesterol (mg/dL)	190.3 ±32.9	189.8 ± 46.1	0.964
Triglyceride (mg/dL)	91.6 ± 44.7	165.3 ± 121.3	0.006
HDL-Cholesterol (mg/dL)	61.6 ± 14.6	45.1 ± 9.8	<0.001
LDL-Cholesterol (mg/dL)	114.5 ± 31.8	118.4 ± 43.8	0.725

AST, aspartate transaminase; ALT, alanine transaminase; GGT, gamma glutamyl peptidase; HDL, high-density lipoprotein; LDL, low-density lipoprotein; * *p* < 0.05 by Student’s *t*-test and chi-square test.

## Data Availability

The data that support the findings of this study are available from the corresponding author, D.W.J., upon reasonable request.
